# Collective directional movement and the perception of social cohesion

**DOI:** 10.1111/bjso.12361

**Published:** 2020-01-03

**Authors:** Stuart Wilson, Jamal K. Mansour

**Affiliations:** ^1^ Division of Psychology, Sociology & Education Queen Margaret University Edinburgh UK

**Keywords:** behavioural coordination, collective action, coordinated action, coordinated movement, directional movement, social cohesion, social perception

## Abstract

We argue that perceivers associate collective directional movement – groups moving from one place to the next – with higher levels of social cohesion. Study 1 shows that pairs are rated as being more cohesive when described as engaging in directional movement compared to non‐directional activities. Study 2 replicates this finding using film clips. Study 3 reveals that the proximity of directionally moving dyads is a better predictor of perceived cohesion than behavioural synchrony. Study 4 replicates the original finding and reveals that perceptions of common fate and shared goals both contribute to the effect, with the former having more predictive power than the latter. We suggest that collective directional movement is an invariant part of social environments and is utilized by perceivers to make inferences about social dynamics.

## Background

Several years ago, whilst travelling alone in Europe, one of us unexpectedly found himself walking down a street alongside a large protest march. Not being familiar with the local language, the chants, shouts, and slogans scrawled on banners were of no help in determining the reason for the group’s apparent discontent. What was very clear, however, was that this was a unified group of people who shared a common purpose. Despite an unsuccessful struggle to infer what that common purpose actually was, what was *not* difficult to infer was that this group, behaving in this way, could reasonably be considered a cohesive entity. This paper is about how certain cues facilitate these kinds of social inferences.

The act of moving from one place to another with others is arguably one of the most ancient and fundamental of all social behaviours. Mobile organisms face a number of potential risks as they move from A to B, and collective movement is one strategy (among many) that has emerged in the biological world to mitigate these risks (Boinski & Garber, 2000). For species that are highly social and dependent upon collaboration, banding together in groups to traverse shared environments can offer a multitude of benefits. Both the modern world and the historical record are replete with examples of humans travelling together, from one place to the next, for all sorts of reasons. It is, we suggest, one of the defining features of human social life and is one of the few social behaviours that connects the first groups of bipedal apes who got together to collectively hunt for prey, seek fresh pastures, or travel to the moon. Each of these activities involved a group of individuals moving from one place to another place, sharing a common fate, with significantly overlapping goals. Somewhere between the innovations of the early hominids and the modern‐day interplanetary explorers we find most other humans, forever repeating a familiar pattern of behaviour: moving as part of a group from one place to another. This is what we are calling *collective directional movement*.

Recently, Wilson, Bassiou, Denli, Dolan, and Watson (2018) defined collective directional movement as an adaptive social behaviour that is notable for the interdependence that it entails between those engaged in it. Interdependence between the constituent parts of a group—or common fate—has long been linked to group cohesion (Campbell, 1958). We define *cohesion* as the prosocial sentiments and feelings of togetherness that exist between members of a group. Such sentiments may take the form of experienced social closeness, often described in such terms as camaraderie and rapport. Members of cohesive groups will trust one another, experience a bond of friendship, and be motivated to act in each other’s interests.

Wilson *et al. *(2018) suggested that the psychological mechanisms governing social bonding are sensitive to certain kinds of group activity. They characterized collective directional movement as a collaborative social behaviour that entails interdependence between participants, and from this they derived predictions about how it might influence first‐person ratings of cohesion. The data from their five studies strongly suggested that engaging in (or imagining engaging in) collective directional movement is associated with higher self‐reported cohesion compared to engaging in (or imagining engaging in) static activities.

With the current studies, we investigated the relationship between collective directional movement and social cohesion from a third‐person perspective. We justify this in the following way. As Wilson *et al. *(2018) point out, the utility of collective directional movement as a behaviour has made it a ubiquitous part of social life. Indeed, one could argue that it represents an invariant feature of human social environments, in the sense that it is found in every society that we know about. As Wilson et al. have shown, participating in collective directional movement promotes social cohesion. This leads us, tentatively, to suggest that the relationship between collective directional movement and social cohesion is recurrent and statistically reliable in social ecologies, both over short timescales and over extended evolutionary time. We therefore propose that collective directional movement represents an invariant part of the social landscape (it is common enough to be seen in all social settings), which, in turn, serves as a reliable indicator of the social dynamics of the individuals engaging in it (based on Wilson et al.’s findings and on the observation that travelling groups will tend to be cohesive more often than not due to the importance of cohesion for successful collaborative activities; see Mullen & Copper, 1994).

Environmental invariances represent potential targets for selection processes when the invariant feature is of adaptive relevance (Barrett, 2015). In the physical world, for example, the fundamental properties of solid objects (e.g., edges and contours) are environmentally invariant. Given that it is adaptive for organisms to make reliable inferences about things in their physical environment, selection processes led to the emergence of perceptual systems that exploit the invariant features of physical objects (e.g., Marr, 1982). This logic is grounded in an information‐processing approach to perception, which allows us to postulate that any input/output mechanisms governing inferences about the world will use as inputs any relevant features of the environment that were reliably present over evolutionary time. We can also apply this reasoning to social perception. If certain social phenomena are recurrent, salient, and information‐rich (to social perceivers), then we might expect mechanisms to develop that exploit these invariant properties, allowing for reliable social inferences to be made (see Haselton & Funder 2006). In relation to this, Pietraszewski et al. (2015) argue that humans possess an *alliance detection system* which ‘carries out the functions of attending to who is allied with whom […] by (1) monitoring for patterns of coordination, cooperation, and competition out in the world, and (2) extracting any cues from the environment (such as location, dress, proximity, shared knowledge, etc.) that happen to correlate with these behaviours, whether these are signalled intentionally or unintentionally’ (p. 25). We suggest that information relating to a group moving together in the same direction will serve as input to any system that functions to extract cues from the environment about potential social alliances.

It is worth stating at this point what we consider to be the constituent features of collective directional movement, how we think these features might influence social judgements, and which of these will be addressed in the current studies. Primarily, there is the *directional* component. All participants engaged in collective directional movement must necessarily be travelling in the same direction. We propose that this in itself provides observers with important information about the nature of the relationship between the individuals in the group. Our first two studies investigate whether collective activities that are either described (Study 1) or perceived (Study 2) as being directional in nature are associated with higher ratings of perceived cohesion compared to control activities that lack any directional information.

Apart from the directional component, there are two other features of collective directional movement that are likely to influence social judgements when encountered in the visual domain: *proximity* and *behavioural coordination*. When groups of individuals travel in the same direction, they usually do so in close proximity to each other. It is possible to imagine a collaborative group that successfully achieves its goal by travelling in the same direction whilst varying their proximity, such as that seen in pincer movements (see also Jacobs, 2010), but, more often than not, members of directionally moving groups will travel close to each other. The proximity of a group’s constituent parts has been linked to how that group is perceived (Campbell, 1958), and it has been hypothesized as an ostensible input to an alliance detection system (Pietraszewski *et al*, 2015). Given our definition of collective directional movement (a group travelling from one place to another), we suggest that proximity might be an important predictor of social cohesion in such groups. This is explored in Study 3.

Similarly, in order to move together in the same direction, members of a travelling group need to achieve a minimum degree of behavioural coordination. Behavioural coordination can be of different types—from those in which the participants move in perfect synchrony to those in which the coordination is much less structured. The effects of behavioural coordination on perceptions of social dynamics have been extensively investigated (e.g., Ip *et al*, 2006; Lakens, 2010; Lakens & Stel, 2011; Wilson & Gos, 2019). The consistent finding is that perceivers associate increased behavioural synchrony with higher levels of social togetherness. We might, therefore, predict the same to be true for travelling groups (which can also take different forms, ranging from the close synchronization of military display marchers to any number of more casual formats requiring only a minimal level of coordinated action). We study the effect of behavioural coordination on perceived cohesion in Study 3.

The act of travelling from location A to location B suggests that those involved will be interdependent, sharing a common fate during the journey (see Campbell, 1958, for a seminal discussion on the link between common fate and social perception; see Wilson *et al.*, 2018, for a discussion on how collective directional movement implies interdependence). It is difficult to conceptualize a travelling group that does not share a common fate, so we argue that this is an inherent feature of the activity and a promising candidate for explaining how such activities might influence social perception. Study 4 measures perceived common fate and investigates how it contributes to perceived cohesion for both directional and non‐directional activities.

A further potential feature emerges from our definition of collective directional movement as a deliberate activity undertaken by a group. This implies that the group members share a common goal, to some extent (see Lickel, Hamilton, & Sherman, 2001, for a discussion on the link between shared goals and social perception). The most basic goal implied is that of successfully traversing a shared environment. Inferences about a travelling group’s goal(s), however, will usually incorporate other information about the purpose for the group’s journey, and so may be more complex than inferences about the group’s common fate. The relationship between perceptions of common goals and cohesion ratings for directional and non‐directional activities is explored in Study 4.

In summary, the studies that follow were designed to test the basic hypothesis that collective directional movement predicts perceptions of social cohesion, and to investigate the nature of this phenomenon.

## STUDY 1

In Study 1, participants were asked to rate the social cohesion of pairs of individuals in two conditions: directional and non‐directional. We predicted that cohesion ratings would be highest when the pair being rated was described as engaging in directional movement. Prior to data collection, institutional ethical approval was granted for the current study and for the other studies described in this paper. For all studies, participants provided informed consent.

## Method

Materials and data for all studies can be accessed at https://osf.io/d6s9c/

Participants were asked to read descriptions of two pairs of people. Each description told of an activity that each pair does together regularly. One of these activities involved directional movement and the other did not.

### Participants and design

Participants (*N* = 62) took part (52 females; *M*_age_ = 23) voluntarily at the beginning of an induction class. Sample size was based on the observation that previous research (Lakens & Stel, 2011; Wilson *et al.*, 2018) investigating similar phenomena has reported medium effect sizes. A 2 × 2 mixed design was employed. Each participant rated how cohesive they perceived two pairs of individuals to be under two conditions: directional movement and non‐directional. This was the repeated‐measures component, and we predicted higher cohesion ratings in the directional movement condition than in the non‐directional condition. The between‐groups component comprised two different versions of the scenarios presented to participants. All participants saw both a ‘running’ scenario and a ‘socializing’ scenario. For each of these scenarios, directional and non‐directional versions were created and participants either responded to running (directional)/socializing (non‐directional) or running (non‐directional)/socializing (directional). If the directional version is associated with greater perceived cohesion, it suggests that directional movement is having an influence beyond what is currently understood about how collective movement influences social perception (e.g., Ip *et al.*, 2006).

### Materials and procedure

After reading an information sheet and signing a consent form, participants were issued with a response booklet (see Appendix S1). The experimenter then provided a brief verbal introduction to the paradigm and explained the task. Participants were invited to work through the response booklet at their own pace. After reading a short introduction, participants were presented with a description of two people. The people described were either called John & James or Sarah & Sue (the pairs of names that were attached to each description were counterbalanced, as was the order that they appeared in). Above the description of the two people was the instruction: ‘as you read, try to imagine the people described and create a vivid mental picture about what they might be like’. Underneath the description was printed: ‘Please take a moment to think about [John & James/Sarah & Sue] and what kind of relationship they might have’.

Each participant saw both a *running* description and a *socializing* description. The descriptions are reproduced below:

*Running (directional)*: [John & James/Sarah & Sue] are the same age. Every Thursday night for the last year they have ran side‐by‐side together on the gently rolling hills of their local town.
*Running (non‐directional)*: [John & James/Sarah & Sue] are the same age. Every Thursday night for the last year they have ran side‐by‐side together on the treadmill running machines at their local gym.
*Socializing (directional)*: [John & James/Sarah & Sue] are the same age. Every Thursday night for the last year they have gone for a walk together.
*Socializing (non‐directional)*: [John & James/Sarah & Sue] are the same age. Every Thursday night for the last year they have gone for a coffee together.

After turning the page, participants were asked to provide a brief written summary of the social scenario that they had just seen. This was done as an engagement check to ensure that participants had paid sufficient attention to the scenario and to allow identification of instances whereby the imagined scenario was discrepant with the presented scenario (e.g., if someone added extra details that changed the scenario in a significant way). After each scenario, participants completed a 10‐item measure of perceived cohesion (derived from the measure used by Wilson *et al*, 2018; see Appendix S1). Responses were on a 7‐point Likert‐style scale and were worded in the following way: ‘Based on the description you have just read, how much trust do you think exists between [John & James/Sarah & Sue]?’ (1 = *They do not trust each other at all*; 7 = *They trust each other completely*).

Items were created that reflected characteristics of cohesion, as per our definition earlier. These included measures relating to prosocial sentiment and social closeness. Items asked about trust between the described pair, the closeness of the relationship between the described pair, levels of bonding, shared humour, camaraderie, friendship, rapport, cooperation, enjoyment of the described activity, and the likelihood of collaborating in the future (definitions of camaraderie and rapport were provided alongside those terms).

It was requested that responses to the items be based on the scenario that the participants had just imagined. After completing the cohesion measure, participants then turned the page to read the second scenario and rated it in the same way, after which they were thanked and debriefed.

## Results and discussion

Six participants were removed from the analysis based on the engagement check. Two had failed to complete the second half of the booklet and the rest had expanded beyond the scenario provided when asked to recount the description. Although there were five participants with mean cohesion scores that were outliers in one of the conditions, excluding these did not make a difference to the results, so they are included. The cohesion instrument showed good internal consistency (Cronbach’s α = .92).

Descriptive statistics can be found in Table 1. As can be seen from inspection of the means, when participants considered pairs described as engaging in directional movement, they rated them as being more cohesive compared to their ratings of pairs described engaging in a non‐directional way. This was true for both activities.

**Table 1:** Study 1 Mean cohesion ratings and 95% confidence intervals (per condition and per stimulus item)

	Mean cohesion (95% CI)
Directional (overall)	5.76 (5.54, 5.97)
Running (hills)	5.80 (5.48, 6.13)
Socializing (walk)	5.72 (5.41, 6.03)
Non‐directional (Overall)	5.03 (4.76, 5.31)
Running (gym)	4.82 (4.36, 5.27)
Socializing (coffee)	5.30 (5.05, 5.55)
Running (Overall)	5.26 (4.94, 5.57)
Socializing (Overall)	5.53 (5.33, 5.74)

To analyse the data, we employed linear mixed‐effects modelling, using cohesion as the dependent variable. This allowed us to incorporate variance at the participant level (see Judd, Westfall & Kenny, 2012) before assessing the contribution of the fixed effects of movement condition (directional/non‐directional) and activity (running/socializing). Although we did not hypothesize any differences between the two activities, we included this factor in the analyses in order to obtain a fuller understanding of our data.

We used the *lmer()* function of the *lme4* package in R (Bates, Maechler, Bolker & Walker, 2015; R Core Team, 2014). When comparing models, we employed the likelihood ratio test, which compares models on a chi‐square distribution (Hox, 2010). We also report the Akaike information criterion (AIC) and the Bayesian information criterion (BIC). To construct the models, we used the maximum likelihood (ML) method of estimation to facilitate model comparisons. Following Field (2009), we built models by (normally) adding one additional predictor to our previous preferred model and comparing these two models.

We began by creating an intercept‐only model (intercept = 5.40, *SE* = .09, AIC = 319.69, BIC = 325.13, *df* = 2) to which we added participants as a random effect (Model 1: AIC = 320.80, BIC = 328.95, *df* = 3). Adding participants as a random factor did not significantly improve the fit over the intercept‐only model, *χ* ^2^(1) = 0.89, *p* = .345, but we kept this factor in our models when making comparisons with the fixed effects as including it better reflects the structure of our data set.

We then added movement condition (directional/non‐directional) as a fixed effect (Model 2: AIC = 302.43, BIC = 313.31, *df* = 4). Adding this significantly improved fit compared to Model 1, *χ* ^2^(1) = 20.37, *p* < .001. To investigate whether the type of activity described by the scenarios (running/socializing) had an independent effect, we added this factor as a fixed effect to Model 1 (Model 3: AIC = 320.24, BIC = 331.11, *df* = 4) and compared it to Model 1. This did not result in a better fit, *χ* ^2^(1) = 2.56, *p* = .110. We next created a model that included the main effects of movement condition (directional/non‐directional) and activity, as well as their interaction (Model 4: AIC = 302.42, BIC = 318.73, *df* = 6). We compared this model to the best‐fitting model (Model 2) and found that fit was not improved, *χ* ^2^(2) = 4.01, *p* = .135. The best‐fitting model, therefore, was Model 2, suggesting that cohesion scores were primarily influenced by movement condition.

These results support the hypothesis that directional movement is associated with social cohesion. It seems that the simple act of moving through space together suggests a greater degree of social cohesion between actors than do non‐directional activities. This hypothesis is tested further in Study 2.

## STUDY 2

In Study 1, we found evidence that collective directional movement is associated with higher levels of perceived cohesion when participants imagined others engaged in social behaviours. In the second study, this hypothesis is extended into the perceptual domain by showing participants short video clips of groups either engaged in directional movement or interacting in a non‐directional manner, and having the groups rated for cohesiveness. We predicted that groups of individuals would be rated as more cohesive when seen engaging in directional movement.

## Method

### Participants and design

Participants (*N* = 50; 35 females; *M*_age_ = 27) were volunteers who responded to email requests for participants to take part in a study on social judgements and had not taken part in the previous study. A repeated‐measures design was employed in which participants rated ten video clips depicting groups of people either moving together in the same direction (the directional condition) or not (the non‐directional condition).

### Materials and procedure

A programme was created that displayed short film clips (without sound) and administered the cohesion instrument after each one. The clips were obtained from a wide variety of sources, from TV drama serials from the 1970s to recent documentaries on current military conflicts. Care was taken not to select clips that may have been familiar to the sample (e.g., clips from well‐known TV shows and movies were excluded in favour of obscure clips and/or clips depicting people likely to be unknown to the participants). The intention was to obtain a selection of scenarios that varied in different ways with the stipulation that they all depicted people who were either moving together in the same direction (the directional movement condition) or interacting in a way that did not involve directional movement (the non‐directional condition). To achieve this, pairs of clips were sought that depicted the same people, wearing the same clothes, behaving in the same way towards each other, in each of the two conditions: directional movement and non‐directional interaction. Finding such clips was surprisingly easy because in many instances where footage exists of people travelling together, there also exists footage of those same people not travelling together either at the beginning or at the end of their journey (or, in some cases, during the journey when they stopped for some reason). Each pair of clips (directional and non‐directional) was edited to be the same length, and care was taken to ensure that the emotions and behaviours displayed in each clip were similar and neutral. The clips are described in Appendix A (which also includes the respective means for each clip on the cohesion measure). The measure of cohesion consisted of the same ten items used in Study 1 (see also the Appendix S1).

On arrival, participants read an information sheet and signed a consent form. The research was described to participants as being on social perception and social judgements. After listening to a brief account of the task, the participants were then told that they would be shown a series of video clips of different lengths, all showing groups of two or more people. It was explained that these would be played without sound and that after each one they would be asked to make judgements about the nature of the relationship between the people in the clips. Each participant viewed and rated ten clips (five in the directional movement condition and five in the non‐directional interaction condition), with each clip being one of the pairs described in Appendix A. Clips were presented in a random order. After rating all of the clips, participants were thanked and debriefed.

## Results and discussion

Cohesion scores for each clip were calculated by taking the average of all the responses on the ten‐item cohesion measure. The cohesion instrument was again found to show good internal consistency (Cronbach’s α = .95). Descriptive statistics can be found in Table 2, which shows that individuals seen engaging in directional movement were rated as more cohesive than when they were seen in a non‐directional interaction.

**Table 2:** Study 2 mean cohesion ratings (95% confidence intervals)

	Mean cohesion (95% CI)
Directional	5.05 (4.92, 5.18)
Non‐directional	3.97 (3.80, 4.14)

We again used linear mixed‐effects modelling in R using the *lmer*() function. The inclusion of outliers did not change the overall results, so we report the analyses with them kept in. We began by creating an intercept‐only model (intercept = 4.51, *SE* = .06, AIC = 1,707.2, BIC = 1,715.6, *df* = 2) to which we added both participants and stimulus items as random effects to create Model 1 (AIC = 1,513, BIC = 1,529.9, *df* = 4), which was a significantly better model compared to the intercept‐only model (*χ* ^2^(2) = 198.13, *p* < .001). To this model, we added movement condition (directional/non‐directional) as a fixed effect (Model 2: AIC = 1,504.3, BIC = 1,525.4, *df* = 5). Adding the dichotomous variable of condition significantly improved fit compared to Model 1, *χ* ^2^(1) = 10.71, *p* = .001.

Again, the hypothesis was supported, this time by utilizing perceptual stimuli. As in the previous study, behaviours indicating directional movement were found to be associated with significantly more perceived cohesion compared to non‐directional interactions. The next study aimed to further investigate the relationship between collective directional movement and social cohesion by examining the contribution of proximity and synchrony. It will also address concerns about potential confounds arising from our use of real‐life clips of social interactions in Study 2.

## STUDY 3

The results of the previous study suggest that viewing others engaged in directional movement leads to those others being perceived as being particularly socially cohesive. However, it is possible that there are hidden variables that have been overlooked. For example, there could have been other social cues present in the clips that might have given the impression of unity/disunity but which had nothing to do with whether the participants were moving together or not. Additionally, the clips used in Study 2 to depict directional movement necessarily showed the groups engaged in coordinated movement (i.e., they were moving in the same direction) *and* proximal movement (i.e., they were doing so in close proximity to each other). Given that one can easily envisage social situations in which these two features of behaviour are independent, it seemed pertinent to investigate what factors influence perceptions of cohesion when individuals are seen to be moving together in the same direction. In order to investigate the roles of proximity and synchrony, Study 3 did not compare a directional movement condition with a non‐directional condition. Instead, pairs of geometric shapes were seen moving across a screen at the same pace. The dimensions on which the pairs varied were proximity and synchrony. The pairs displaying low proximity and low synchrony as they travelled across the screen were expected to be perceived as being the least cohesive, and the pairs displaying high proximity and high synchrony were expected to be perceived as being most cohesive.

## Method

### Participants and design

Participants (*N* = 27; 23 females; M_age_ = 25) were recruited via a participant database maintained by our division and classroom appeals. A repeated‐measures design was employed in which two cohesion, proximity, and synchrony ratings for pairs of dots moving across a screen were recorded for each of the four conditions that derive from factorially crossing proximity (low, high) with synchrony (low, high).

### Materials and procedure

We created eight short animations, each depicting a red dot and a blue dot moving across the screen from left to right. In each clip, the dots started next to each other on the left‐hand side of the screen and then, 20 s later, ended up next to each other on the right‐hand side of the screen. Two animations were created for each of the four conditions. For the high proximity animations, the two dots stayed close to each other for the entire duration; for low proximity animations, one dot moved to the top of the screen and one dot moved to the bottom of the screen, before coming together again at the end. For the high synchrony animations, the two dots both behaved in exactly the same ways during the 20 s (i.e., their behaviour was perfectly synchronized); for the low synchrony animations, the two dots behaved in completely different ways during the 20 s (i.e., their behaviour was not synchronized at all, except for the fact that they were travelling towards the same location). As the task was somewhat unusual (assigning sociality to geometric shapes), a brief orienting task was also administered after participants read the information sheet and provided consent. This involved providing participants with a short introduction to the field of social perception and then showing them a brief segment (10 s) from the clip used by Heider and Simmel (1944) in which geometric shapes are perceived as social actors. We chose a section of this clip that clearly showed one of the geometric shapes displaying fear of another geometric shape and asked participants to answer a question about the social relationship between the shapes in the clip (the answer to which was deliberately straightforward given the specific selection of the segment). After completing the orienting task, participants were told that the main task would involve them making social judgements about animated geometric shapes. After having had the chance to ask questions, participants proceeded to the main task. The order that the animations were presented in was randomized. After each clip, participants completed a version of the 10‐item cohesion instrument used in the previous studies. Additionally, participants rated the synchrony and proximity of each pair of shapes. After providing ratings for the eight clips, participants were thanked and debriefed.

## Results and discussion

The internal consistency of the cohesion measure was again found to be high (Cronbach’s α = .98). Outliers were included as they were found not to make a difference to the outcomes of the analyses. Descriptive statistics can be found in Table 3. Inspection of the means suggests that both proximity and synchrony contribute to perceived cohesion, with proximity appearing to have the largest influence.

**Table 3:** Study 3 mean cohesion, synchrony, and proximity ratings (95% confidence intervals)

	Mean cohesion (95% CI)	Mean proximity (95% CI)	Mean synchrony (95% CI)
High Proximity/High Synchrony	6.25 (6.01, 6.50)	6.80 (6.61, 6.98)	6.91 (6.81, 7.00)
High Proximity/Low Synchrony	4.91 (4.46, 5.35)	5.98 (5.63, 6.33)	4.31 (3.77, 4.86)
Low Proximity/High Synchrony	3.92 (3.49, 4.35)	2.87 (2.51, 3.23)	5.72 (5.28, 6.16)
Low Proximity/Low Synchrony	2.36 (2.01, 2.71)	1.85 (1.52, 2.18)	1.89 (1.60, 2.18)

Again, we constructed an intercept‐only model (intercept = 4.36, *SE* = .13, AIC = 909.52, BIC = 916.27, *df* = 2) to which we added both participants and stimulus items as random effects (Model 1; AIC = 780.08, BIC = 793.58, *df* = 4), significantly improving the model fit compared to the intercept‐only model, *χ* ^2^(2) = 133.44, *p* < .001. To Model 1, we added the synchrony ratings (Model 2; AIC = 742.09, BIC = 758.96, *df* = 5), again significantly improving the fit, *χ* ^2^(1) = 39.99, *p* < .001.

Model 3 replaced the synchrony ratings in Model 2 with the proximity ratings (AIC = 746.38, BIC = 763.25, *df* = 5), and we compared this new model with Model 1. Proximity ratings also made an independent contribution to model fit, *χ* ^2^(1) = 35.70 *p* < .001. We then built a model that added both the proximity ratings and the synchrony ratings as covariates to Model 1 (Model 4; AIC = 704.80, BIC = 725.05, *df* = 6) and found that this model was a better fit to our data than both Model 2 (the model with synchrony as a single covariate), *χ* ^2^(1) = 39.29, *p* < .001, and Model 3 (the model with proximity as a single covariate), *χ* ^2^(1) = 43.58, *p* < .001.

Finally, we built a model (Model 5) that included all of the terms from Model 4 and added the interaction of synchrony and proximity (AIC = 706.63, BIC = 730.26, *df* = 7). This did not improve fit compared to Model 4, *χ* ^2^(1) = 0.17, *p* = .683. The best‐fitting model, therefore, was Model 4, in which ratings of proximity and synchrony make independent contributions. The picture that emerges from these data suggests that, for entities moving in the same direction, both behavioural synchrony and proximity are important in determining how cohesive the entities are perceived to be, with proximity appearing to be the more important predictor of the two (see Table 3).

## STUDY 4

As discussed in the introduction, most groups engaging in directional movement will share a common fate during their journey. This raises the possibility that perceivers may utilize judgements about the group’s fate when estimating social dynamics. Similarly, many such groups will also share goals, although as we stated previously, the perception of shared goals is likely to involve a more complex series of inferences compared to the perception of common fate, and so may not be as strongly linked to the perception of social cohesion as we expect perceptions of common fate to be.

The purpose of Study 4 was to replicate the effect of collective directional movement on perceived cohesion using a new textual manipulation and to investigate whether the effect is due to perceptions of common fate and/or shared goals. Using a different text‐based scenario to what was used in Study 1 allows us to be more confident that our effect is generalizable to other instances of implied directional movement. Both of the scenarios used in Study 1 described recurring activities (the individuals were described as meeting once a week), whereas the scenarios used in the current study involved groups of strangers engaging in a single activity that takes place over an extended period of time. This should provide us with a picture of how directional information influences perceptions of group cohesion that is not influenced by considerations of the existing relationship between the individuals described.

## Method

### Participants and design

Participants (*N* = 128; 64 females; *M*_age_ = 28) were recruited via an online recruitment platform (Prolific Academic), were fluent English speakers, and were paid the equivalent of £5/hour. A repeated‐measures design was employed in which participants were asked to read and reflect upon descriptions of two groups, one of which was described as engaging in directional movement and one of which was not. Participants rated each group on cohesion, common fate, and shared goals.

### Materials and procedure

After providing informed consent and reading instructions explaining the task (see Appendix S1), participants read and rated two scenarios, one after the other. The order of presentation was counterbalanced across the sample. The scenarios are reproduced below and were based on similar items successfully used by Wilson *et al. *(2018).

*Directional*: We would like you to imagine a group of eight people travelling on an important journey. The journey will take three days. None of the group knew each other before they began their journey. All members of the group volunteered to go on the journey.

*Non‐directional*: We would like you to imagine a group of eight people attending an important event. The event will last for three days. None of the group knew each other before they met at the event. All members of the group volunteered to attend the event.

After reading the description, participants were instructed not to proceed until they had a clear image in their heads of what the group might be like during the activity described. At this stage, we presented four questions designed as attention checks to make sure that the scenarios had been read and understood. These asked about basic facts that were part of the described scenario and an error or omission on any of these questions resulted in exclusion from the analyses. A further question asked participants to state two things that they imagined happening during the time that the group was together. Failure to provide two responses for each scenario resulted in exclusion. If the responses provided indicated that a scenario had been misconstrued, or if an imagined aspect was deemed inappropriate for the condition (e.g., mentioning a directional movement activity in the static condition), then the data for that participant were excluded. We put in place more stringent attention checks and exclusion criteria for Study 4 because, unlike the prior experiments, this study was conducted online. The exclusion criteria for this study were pre‐registered and can be viewed at https://osf.io/xmtuc/?view_only=df7a2d8556fd4464a05562b6a8fc0f6b. The measured variables were the same 10‐item cohesion measure used in previous studies along with questions asking about perceptions of common fate and shared goals. These were worded in the following way: ‘Based on how you imagined the scenario, to what extent would you say the group members shared the same goals whilst they were travelling/at the event?’ (1 = *Their goals did not overlap at all*; 7 = *Their goals overlapped entirely*); ‘“Common fate” is the extent to which an individual's fate is linked to the rest of the group. Based on how you imagined the scenario, to what extent would you say that the group members shared a common fate during the journey/event?’ (1 = *The fate of each individual was entirely separate to the rest of the group*; 7 = *The fate of each individual was entirely linked to the rest of the group*). All responses were provided on a 7‐point Likert‐style scale.

Once participants had rated the scenarios, we asked some final questions as a general engagement check (e.g., To what extent did you hurry through this survey? 1 = *Not at all*; 7 = *Very Much*) and provided participants with an opportunity to tell us anything they thought we needed to know.

## Results and discussion

Twenty participants were excluded from the analysis. Six were excluded because they mentioned directional movement in their description of the non‐directional scenario; seven due to inappropriate or incongruous descriptions of the imagined scenario; six due to incomplete or incorrect responses to the attention checks; and one due to the participant reporting to the researcher that they thought they had done the same condition twice.

The internal consistency of the cohesion measure was again found to be high (Cronbach’s α = .91). There were six participants who had at least one of their mean cohesion scores more the two standard deviations from the mean of the condition. Analyses were performed both with and without these participants, and it was found to make little difference to the overall results. We note where there was a difference where appropriate. The engagement checks showed that participants were engaged and a composite measure of engagement was not found to be related to the dependent variable, and so will not be considered further.

Descriptive statistics can be found in Table 4 below. They suggest that all measured variables were rated higher, on average, in the directional movement (‘journey’) condition compared to the non‐directional (‘event’) condition.

**Table 4:** Study 4 mean ratings and 95% confidence intervals for both conditions

	Mean cohesion (95% CI)	Mean common fate (95% CI)	Mean shared goals (95%CI)
Journey (directional)	4.27 (4.06, 4.47)	4.56 (4.24, 4.88)	5.10 (4.85, 5.35)
Event (non‐directional)	3.82 (3.62, 4.01)	3.71 (3.39, 4.03)	4.83 (4.54, 5.12)

As with the previous analyses, our dependent measure was the averaged cohesion score. We began by creating an intercept‐only model (intercept = 4.04, *SE* = .07, AIC = 582.52, BIC = 589.12, *df* = 2) to which we added participants as a random effect (Model 1: AIC = 576.55, BIC = 586.45, *df* = 3). This was found to be a significant improvement over the intercept‐only model, *χ* ^2^(1) = 7.97, *p* = .005. To Model 1, we added condition (directional/non‐directional) as a fixed effect (Model 2: AIC = 564.33, BIC = 577.53, *df* = 4). Adding this effect significantly improved fit compared to Model 1, *χ* ^2^(1) = 14.22, *p* < .001, supporting our basic prediction.

We next investigated the contributions of our covariates. Model 3 added the ratings of common fate (AIC = 524.97, BIC = 541.46, *df* = 5). When compared to Model 2, the model with common fate was a significantly better predictor of cohesion, *χ* ^2^(1) = 41.36, *p* < .001. We then looked for an interaction between common fate ratings and condition (Model 4: AIC = 525.29, BIC = 545.08, *df* = 6), but this model did not improve fit over Model 3, suggesting that there was no interaction, *χ* ^2^(1) = 1.68, *p* = .195.

Model 5 investigated the independent contribution of the shared goal ratings (AIC = 546.95, BIC = 563.44, *df* = 5). We compared this model to our condition‐only model (Model 2), finding that the model that included the shared goal ratings was a significant improvement, *χ* ^2^(1) = 19.38, *p* < .001. Adding an interaction term between the shared goal ratings and condition (Model 6; AIC = 548.71, BIC = 568.50, *df* = 6) did not improve fit over Model 5, suggesting no interaction between the shared goal ratings and condition in terms of their ability to predict cohesion, *χ* ^2^(1) = 0.235, *p* = .628.

Model 7 included the ratings for both common fate and shared goals (AIC = 523.23, BIC = 543.02, *df* = 6). This was found to be a better fit than Model 5 (shared goal ratings only), *χ* ^2^(1) = 25.72, *p* < .001. The improvement over Model 3 (common fate ratings only) was marginally significant; *χ* ^2^(1) = 3.74, *p* = .053. This result suggests that the ratings of common fate may have more explanatory power than the ratings of shared goals (note: when the analyses are performed with outliers on the cohesion measure removed, the difference between Models 7 and 3 becomes more definitively non‐significant, *χ* ^2^(1) = 3.31, *p* = .069.

Model 8 built on Model 7 by adding a term for the interaction between common fate and shared goals (AIC = 524.60, BIC = 547.69, *df* = 7), but this was not a significant improvement over Model 7, suggesting that these covariates make independent contributions, *χ* ^2^(1) = 0.63, *p* = .429.

Finally, we compared Model 7 (includes fixed effect of condition, common fate, and shared goals) with a model that comprised only common fate and shared goals (Model 9; AIC = 525.42, BIC = 541.92, *df* = 5). This was done to assess whether the covariates could account for the cohesion ratings without taking the condition into account. The model that included condition (i.e., Model 7) was a better fit to the data, *χ* ^2^(1) = 4.20, *p* = .04 (note: when the analyses are performed without participants who were outliers on the cohesion measure, this difference becomes more pronounced, *χ* ^2^(1) = 11.53, *p* < .001. This suggests that, although both covariates make independent contributions towards explaining the variance in cohesion ratings (with the common fate ratings being the more dominant predictor of the two), they cannot fully explain the difference in the cohesion ratings between the two experimental conditions.

Although we have demonstrated that cohesion is influenced by directional movement and perceptions of common fate and shared goals, we have not demonstrated that directional movement is related to common fate and/or shared goals. To test for a relationship between directional movement and the perception of common fate/shared goals, we conducted exploratory model tests. We first constructed a null two‐level model predicting common fate (Model 10: AIC = 765.85, BIC = 775.75, *df* = 3) and compared it to a two‐level model with condition as a fixed effect (Model 11: AIC 746.58, BIC = 759.77, *df* = 4). The latter model was a significantly better fit to the data, *χ* ^2^(1) = 21.28, *p* < .001, indicating that directional movement predicts higher ratings of common fate compared to the non‐directional condition (see Table 4).

We did the same analysis with perceptions of shared goals as the dependent measure. The null two‐level model (Model 12: AIC = 688.65, BIC = 698.55, *df* = 3) fits the data as well as the model with condition as a fixed effect (Model 13: AIC = 687.92, BIC 701.12, *df* = 4), *χ* ^2^(1) = 2.73, *p* = .099, suggesting that, unlike the ratings for common fate, ratings for shared goals are not different in the directional and non‐directional conditions.

## GENERAL DISCUSSION

The results from four studies, using a variety of methodologies, generally support the hypothesis that groups engaging in directional movement are seen as being more cohesive compared to groups engaged in non‐directional activities. Taken in isolation, each of our studies and methodologies has strengths and weaknesses. This is partly due to the challenges in operationalizing the central constructs and in implementing appropriate control conditions. Identifying suitable control conditions for studies such as these is particularly difficult because it is not obvious what characteristics the non‐directional conditions should have (or not have) in order to allow for a fair comparison with the directional conditions. Taken together, however, the consistent results from our series of studies offer good support for our hypothesis.

Study 1 demonstrated the effect using two social scenarios that were presented textually in both directional and non‐directional formats. Wilson *et al *(2018) found that collective directional movement was associated with higher self‐reported cohesion compared to non‐directional activities. We extend these findings by demonstrating that information about unknown individuals engaging in directional movement together is used by perceivers to make inferences about their ostensible social relationship, and that it is the directional component of the information that influences such judgements. Not only do people feel more cohesive when they have engaged in collective directional movement, but also other people expect them to feel more cohesive. Although it was not a focus of the current work, it is worth mentioning that pairs in the ‘running’ scenarios in Study 1 were rated as being more cohesive when they were described as running together in the hills (directional) compared to on treadmills (non‐directional). This suggests that the effect is not solely due to similarities in movement, and mirrors a finding reported by Wilson *et al *(2018) in relation to first‐person experience. A post hoc test confirmed that the cohesion scores on our two versions of the running scenario were significantly different, *t*(54) = 3.45, *p* = .001, two‐tailed. This is something that should be explicitly addressed in follow‐up work.

Study 2 demonstrated that the effect extends to ratings based on video clips. Aside from replicating the effect in another domain, the data from the second study revealed how robust the effect is. Although the stimulus clips were disparate in terms of the nature of the groups shown, all but one pair conformed to the predicted difference in cohesion ratings between the directional and non‐directional versions (see Appendix A).

Another aim of the current work was to test whether the effects of collective directional movement on perceptions of cohesion could be explained by other factors, such as those relating to the act itself and other potential confounds. Study 3 removed the possibility of confounding social cues by using geometric shapes as stimuli and investigated the respective roles of proximity and behavioural coordination. Both were found to make independent contributions to the variance in cohesion ratings, with proximity being the more dominant of the two. Study 4 replicated the basic effect and revealed that ratings of common fate and shared goals both make independent contributions, with common fate ratings emerging as the better predictor (indeed, common fate ratings were significantly higher in the directional scenario, which was not the case for the shared goal ratings). However, the variance explained by these covariates was insufficient to entirely account for the difference in cohesion scores over the two conditions (directional/non‐directional). This result broadens our understanding of the effects of collective directional movement by suggesting that directional information contributes something to perceptions of cohesion over and above information indicating shared goals and common fate. Future work should aim to clarify what other influences might exist. As previously stated, inferences about the goals of a travelling group’s members may be more complex and heterogeneous than inferences about that group’s common fate. Future studies might want to manipulate perceptions of goal overlap in order to understand its contribution to perceived cohesion, both independently and in conjunction with perceptions of common fate (and any other covarying factors yet to be identified).

Taken together, our findings suggest that extracting information about social dynamics from the movement of groups involves more than what traditional research into this topic has suggested. Our results point to directionality as a feature of dynamic social interactions that should be considered as an important contributing factor alongside what has traditionally been shown to have an influence on such judgements, such as behavioural coordination. Specifically, directional movement that implies a common fate and is undertaken in close proximity appears to be particularly potent as a means to judge social closeness.

Our findings do have limitations. Asking people to make social judgements based on short text descriptions (Studies 1 and 4) may not reflect how such decisions are usually made. There is also likely to be a degree of heterogeneity in the way different people conceptualize the same scenario (this was evident from the short descriptions provided by participants in Study 4). Similarly, there are ecological validity issues when using simple geometric shapes as social stimuli (Study 3). Again, how people engage with these artificial stimuli may be a source of uncontrolled variance, and so we must be careful not to extend the findings from such studies too keenly, as more work needs to be done to understand how the various contributing factors interact with each other and whether the same effects can be demonstrated with different stimulus formats. What our results generally suggest, however, is that information about directionality is taken into consideration over a range of domains when making inferences about social relationships.

Our cohesion instrument, although clearly reliable (see also Wilson *et al.*, 2018), has not been evaluated in any other way, and so this must also be considered a potential limitation of the current work. One aim of future research should be to further validate this instrument, and also to explore how it relates to other relevant variables. Doing so would help establish a broad foundation for the reported phenomena and place it within the wider social perception literature.

Understanding how our results articulate with other phenomena concerning the perception of groups should be a focus for future research. For example, are directionally moving groups seen as being more ‘group‐like’ than other groups, a phenomenon known as entitativity (Lickel, Hamilton, & Sherman, 2001)? If so, what might be the implications for how perceivers prepare to interact with such groups: are they assessed as a potential threat, for example (Hackel, Looser, & Van Bavel, 2014)? Additionally, boundary conditions need to be mapped. Based on what we presently understand about group perception (see Yzerbyt, Judd, & Corneille, 2004, for an overview), we might expect participants to consider the composition of the group (e.g., who the group members are/what their relationship is to each other) and/or the process by which the group coalesced into a unit (e.g., the choices made by the participants and the reasons for those choices) when making judgements about a group’s social dynamics. This is something that future work might manipulate. Similarly, information about the effort involved in participation or sacrifices that individuals make in order to participate may also influence judgements about social dynamics. In relation to this, Lakens and Stel (2011) and Wilson and Gos (2019) both reported data suggesting that social judgements made by observers on the basis of movement dynamics are mediated by inferences about the intentions of the individuals being observed.

Understanding boundary conditions is particularly challenging given that collective directional movement is now a much broader class of social behaviour than at any previous point in human history. We live in an age of mass transit, where technology can transport individuals in ways and over distances that earlier generations would not have been able to imagine. These are very different formats of collective directional movement compared to what humans were engaging in for thousands of years before the advent of such innovations. Where once humans engaging in collective directional movement could reasonably be said to have had some form of social relationship with their travel companions, our recent history has turned this on its head, and humans now regularly engage in such behaviours with strangers. Yet, all versions of mass transit retain some core attributes of the basic behaviour: people get together and move through space, from one place to another. How are our perceptions of social dynamics influenced by the various versions of directional movement that we now implement in modern societies? Does the inevitable common fate of accidental travel companions interact with other social forces? These remain open and empirical questions for future research to investigate.

Given its ubiquity and inherent properties, it would be surprising if collective directional movement was not exploited by mental systems involved in deriving meaning from the world of others, just as visual systems exploit the properties of the physical world. It is a social phenomenon that we see almost everywhere we look, yet the social psychology community has had relatively little to say about it beyond what is known about the links between movement synchrony and socio‐perceptual phenomena (Reddish, Bulbulia, & Fischer, 2014). As such, we hope that the current paper encourages other researchers to take up investigating collective directional movement as a social psychological phenomenon, given that it is one of our most common and familiar social behaviours.

## Conflicts of interest

The authors declare that there is no conflict of interest.

## Supporting information


**Appendix S1.** Materials.

**Code S1.** Materials.

## Appendix A Descriptions of the clips used in Study 2. Figures represent the average score for each clip on the cohesion measure and 95% Confidence Intervals

**Table:**

General description (+ length)	Directional	Non‐Directional
A middle‐aged man and a young man, both dressed smartly in suits, have a discussion (43s)	They walk together at a leisurely pace through a park. They are alone and no others are shown. Cohesion: 5.18 (4.75, 5.62)	They are sitting together at a table in a restaurant. The cameras remain close on the men, and no others are shown during the clip. Cohesion: 3.27 (2.76, 3.78)
Two smartly dressed older men have a discussion (16s)	They walk together, at a slow pace through a busy European city street. The camera remains tight on the men, and other people are seen in the background. Cohesion: 4.77 (4.33, 5.20)	They sit talking together in a bar. There are other people in the bar, and the camera remains focused on the two men talking. Cohesion: 3.87 (3.33, 4.40)
A group of approximately 20 males and females participating in a fitness test (5s)	They are seen running along a beach side by side. There are no others seen who are not part of the group. Cohesion: 5.52 (5.16, 5.88)	They are seen at the end of the run, standing together at the completion point. There are no others seen who are not part of the group. Cohesion: 4.88 (4.43, 5.32)
Three male hunter–gatherers in their 30s/40s (10s)	They walk across a clearance carrying baskets and tools. No others are shown. Cohesion: 5.66 (5.40, 5.91)	They are seen sitting in their dwelling talking. No others are shown. Cohesion: 4.11 (3.52, 4.70)
Four adult male family members living in a small tribal village in Ethiopa (5s)	They walk away from their village along a path in single file. No others are shown. Cohesion: 5.32 (4.93, 5.71)	They are seen sitting in silence outside their hut. No others are shown. Cohesion: 5.51 (5.10, 5.91)
Eight males of varying ages filming their camping trip in the jungle (15s)	They are seen walking through the jungle with their equipment. Some are seen speaking to each other, and others are filming. No others are shown. Cohesion: 4.74 (4.36, 5.12)	They are seen sitting round a camp‐fire underneath a shelter. Some are seen speaking, and others are filming. No others are shown. Cohesion: 3.27 (2.76, 3.78)
Eight females of varying ages filming their camping trip in the jungle (23s)	They are seen making their way through the jungle together. They talk to each other as they walk. No others are shown. Cohesion: 5.46 (5.08, 5.83)	They are seen in a clearing, standing in a circle, discussing with each other which direction they should take. No others are shown. Cohesion: 3.45 (3.09, 3.81)
A group of two males and two females participating in a “10,000 BC Challenge”. They are dressed in animal skins and are carrying sharpened sticks (24s)	They walk together through a thick forest. No others are shown. Cohesion: 4.42 (4.02, 4.82)	They stop at a ravine and are deciding which route to take next. No others are shown. Cohesion: 3.27 (2.77, 3.76)
A group of fifteen female guerilla fighters, filmed from behind (4s)	They are seen walking out of their briefing, and up a narrow path in single file. No others are shown. Cohesion: 4.03 (3.55, 4.52)	They are seen sitting in a small briefing room awaiting the start of a briefing. No others are shown. Cohesion: 3.30 (2.83, 3.77)
Two males and two females (aged in 20s & 30s) walking in Africa (3s)	They walk together across an expansive and empty Savannah. No others are shown. Cohesion: 5.40 (5.09, 5.70)	They are seen standing in their camp preparing for their journey. No others are shown. Cohesion: 4.63 (4.23, 5.04)

## References

[bjso12361-bib-0001] Barrett, H. C. (2015). The shape of thought: How mental adaptations evolve. Oxford, UK: Oxford University Press.

[bjso12361-bib-0002] Bates, D. , Mächler, M. , Bolker, B. , & Walker, S. (2015). Fitting linear mixed‐effects models using lme4. Journal of Statistical Software, 67(1), 1–48. 10.18637/jss.v067.i01

[bjso12361-bib-0003] Boinski, S. , & Garber, P. A. (2000). On the Move: How and why animals travel in groups. Chicago, IL: University of Chicago Press.

[bjso12361-bib-0004] Campbell, D. T. (1958). Common fate, similarity, and other indices of the status of aggregates of persons as social entities. Behavioral Science, 3, 14–25. 10.1002/bs.3830030103

[bjso12361-bib-0005] Field, A. (2009). Discovering statistics using SPSS (and Sex, Drugs, and Rock ‘n’ Roll). London, UK: Sage.

[bjso12361-bib-0006] Hackel, L. M. , Looser, C. E. , & Van Bavel, J. J. (2014). Group membership alters the threshold for mind perception: The role of social identity, collective identification, and intergroup threat. Journal of Experimental Social Psychology, 52, 15–23. 10.1016/j.jesp.2013.12.001

[bjso12361-bib-0007] Haselton, M. G. , & Funder, D. C. (2006). The evolution of accuracy and bias in social judgment In Schaller M., Simpson J. A., & Kenrick D. T. (Eds.), Evolution and social psychology (pp. 15–37), New York, NY: Psychology Press.

[bjso12361-bib-0008] Heider, F. , & Simmel, M. (1944). An experimental study of apparent behaviour. American Journal of Psychology, 57, 243–259. 10.2307/1416950

[bjso12361-bib-0009] Hox, J. (2010). Multilevel analysis: Techniques and applications (2nd ed.). New York, NY: Routledge.

[bjso12361-bib-0010] Ip, G. W. , Chiu, C. , & Wan, C. (2006). Birds of a feather and birds flocking together: Physical versus behavioural cues may lead to trait‐ versus goal‐based group perception. Journal of Personality and Social Psychology, 90, 368–381. 10.1037/0022-3514.90.3.368 16594825

[bjso12361-bib-0011] Jacobs, A. (2010). Group cohesiveness during collective movements: Travelling apart together. Behavioural Processes, 84, 678–680. 10.1016/j.beproc.2010.03.004 20350590

[bjso12361-bib-0012] Judd, C. M. , Westfall, J. , & Kenny, D. A. (2012). Treating stimuli as a random factor in social psychology: A new and comprehensive solution to a pervasive but largely ignored problem. Journal of Personality and Social Psychology, 103(1), 54–69. 10.1037/a0028347 22612667

[bjso12361-bib-0013] Lakens, D. (2010). Movement synchrony and perceived entitativity. Journal of Experimental Social Psychology, 46, 701–708. 10.1016/j.jesp.2010.03.015

[bjso12361-bib-0014] Lakens, D. , & Stel, M. (2011). If they move in sync, they must feel in sync: Movement synchrony leads to attributions of rapport and entitativity. Social Cognition, 29(1), 1–14. 10.1521/soco.2011.29.1.1

[bjso12361-bib-0015] Lickel, B. , Hamilton, D. L. , & Sherman, S. J. (2001). Elements of a lay theory of groups: Types of groups, relational styles, and the perception of group entitativity. Personality and Social Psychology Review, 5, 129–140. 10.1207/S15327957PSPR0502_4

[bjso12361-bib-0016] Marr, D. (1982). Vision. Cambridge, MA: The MIT Press.

[bjso12361-bib-0017] Mullen, B. , & Copper, C. (1994). The relation between group cohesiveness and performance: An integration. Psychological Bulletin, 115, 210–227. 10.1037/0033-2909.115.2.210

[bjso12361-bib-0018] Pietraszewski, D. , Curry, O. S. , Petersen, M. B. , Cosmides, L. , & Tooby, J. (2015). Constituents of political cognition: Race, party politics, and the alliance detection system. Cognition, 140, 24–39. 10.1016/j.cognition.2015.03.007 25867997

[bjso12361-bib-0019] R Core Team (2014). R: A language and environment for statistical computing. Vienna, Austria: R Foundation for Statistical Computing http://www.R-project.org/

[bjso12361-bib-0020] Reddish, P. , Bulbulia, J. , & Fischer, R. (2014). Does synchrony promote generalized prosociality? Religion, Brain and Behavior, 4(1), 3–19. 10.1080/2153599x.2013.764545

[bjso12361-bib-0021] Wilson, S. , Bassiou, E. , Denli, A. , Dolan, L. C. , & Watson, M. (2018). Traveling groups stick together: How collective directional movement influences social cohesion. Evolutionary Psychology, 16(3), 147470491879213 10.1177/1474704918792134 PMC10480943 30071757

[bjso12361-bib-0022] Wilson, S. , & Gos, C. (2019). Perceiving social cohesion: Movement synchrony and task demands both matter. Perception, 48, 316–329. 10.1177/0301006619837878 30871427

[bjso12361-bib-0023] Yzerbyt, V. , Judd, C. M. , & Corneille, O. (Eds.) (2004). The psychology of group perception: Perceived variability, entitativity, and essentialism. New York, NY: Psychology Press.

