# Supplementary material for: Collective directional movement and the perception of social cohesion
Source: Br J Soc Psychol. 2020 Jan 3;59(4):819–38. doi: 10.1111/bjso.12361 (PMC7586976; doi:10.1111/bjso.12361)
Supplement: Supplementary file 1 — Appendix S1. Materials. [file BJSO-59-819-s001.docx]

MATERIALS

Study 1

Thank you for agreeing to take part in our research project. We are investigating how people make quick social judgments & decisions based on limited information.

To study this, we are going to ask you to read short descriptions of two people and then answer some questions about them. We are going to deliberately give you as little information as we can. When answering the questions about them, please try to think about what their relationship might be like based on the information you have been given. Try to create a picture in your mind of what kind of interactions the people might have and what things they might do together based on the information provided.

The first description is in the box below. As you read it, try to imagine the people described and create a vivid mental picture about what their relationship might be like*.*

*<scenario goes here>*

Please take a moment to think about ***Sarah and Sue/James and John /James and John*** and what kind of relationship they might have. Try to imagine their relationship before, during and after the activity described above. Once you have done that, please turn the page and answer some questions. Please do not turn the page until you have a clear image in your head about Sarah and Sue/James and John /James and John’s relationship.

Just so we know you’ve been paying attention, please briefly summarise the description of Sarah and Sue/James and John that you were asked to imagine in the box below (It’s fine to look back if you need to remind yourself, but please don’t add any details - we only want you to recount the main details so we know that you have understood the scenario):

We will now ask you some questions. Please answer them by circling the appropriate number on the scale provided. You should base you answers on the scenario that you have just imagined (it’s fine to look back at the scenario if you need to remind yourself at any point). There are no right or wrong answers so please be as honest and as accurate as you can. All responses are anonymous.

*Based on the information you have been given, how much* ***trust*** *do you think exists between Sarah and Sue/James and John ?*

1 2 3 4 5 6 7

They do not trust each other at all

They trust each other completely

*Based on the information you have been given, how might you describe the* ***relationship*** *between Sarah and Sue/James and John ?*

1 2 3 4 5 6 7

Like “blood relatives”

Like total strangers

*Based on the information you have been given, how would you describe the level of* ***bonding*** *between Sarah and Sue/James and John ?*

1 2 3 4 5 6 7

Not bonded at all

Extremely well bonded

*Based on the information you have been given, how much* ***humour*** *do you think exists between Sarah and Sue/James and John ?*

1 2 3 4 5 6 7

None at all

A lot

*Based on the information you have been given, how much* ***camaraderie*** *do you think exists between Sarah and Sue/James and John ? (*camaraderie = a spirit of familiarity and trust*)*

1 2 3 4 5 6 7

None at all

A lot

*Based on the information you have been given, how much* ***friendship*** *do you think exists between Sarah and Sue/James and John ?*

1 2 3 4 5 6 7

None at all

A lot

*Based on the information you have been given, how much* ***rapport*** *do you think exists between Sarah and Sue/James and John ? (*Rapport *=* a close and harmonious relationship in which the people concerned understand each other's feelings or ideas and communicate well)

1 2 3 4 5 6 7

None at all

A lot

*Based on the information you have been given, how much* ***co-operation*** *do you think would be evident between Sarah and Sue/James and John ?*

1 2 3 4 5 6 7

None at all

A lot

*Based on the information you have been given, please estimate how much Sarah and Sue/James and John* ***enjoy each other’s company***

1 2 3 4 5 6 7

They do not enjoy each other’s company at all

They enjoy each other’s company a lot

*Based on the information you have been given, how likely do you think it is that Sarah and Sue/James and John will* ***work together in the future?***

1 2 3 4 5 6 7

Highly Likely

Not at all likely

Study 2

Thank you for agreeing to take part in this research

In this experiment, you will be shown a series of short film clips. Some of these will only last for a few seconds, others will be longer.

The clips will be shown with no sound.

Please watch each clip carefully.

After watching each clip you will be asked to make judgments about the nature of the relationship between the people in the clips.

We have deliberately made this task difficult because we want to see how people make judgements based on limited information.

There are no right or wrong answers. Just be as honest as you can when reporting your judgements.

Please judge each clip individually and try not to let your judgement be influenced by previous clips

<clips>

*Based on the clip you have just watched, how much* ***trust*** *do you think exists between the people in the clip ?*

1 2 3 4 5 6 7

They do not trust each other at all

They trust each other completely

*Based on the clip you have just watched, how might you describe the* ***relationship*** *between the people in the clip ?*

1 2 3 4 5 6 7

Like “blood relatives”

Like total strangers

*Based on the clip you have just watched, how would you describe the level of* ***bonding*** *between the people in the clip ?*

1 2 3 4 5 6 7

Not bonded at all

Extremely well bonded

*Based on the clip you have just watched, how much* ***humour*** *do you think exists between the people in the clip ?*

1 2 3 4 5 6 7

None at all

A lot

*Based on the clip you have just watched, how much* ***camaraderie*** *do you think exists between the people in the clip ? (*camaraderie = a spirit of familiarity and trust*)*

1 2 3 4 5 6 7

None at all

A lot

*Based on the clip you have just watched, how much* ***friendship*** *do you think exists between the people in the clip ?*

1 2 3 4 5 6 7

None at all

A lot

*Based on the clip you have just watched, how much* ***rapport*** *do you think exists between the people in the clip ? (*Rapport *=* a close and harmonious relationship in which the people concerned understand each other's feelings or ideas and communicate well)

1 2 3 4 5 6 7

None at all

A lot

*Based on the clip you have just watched, how much* ***co-operation*** *do you think would be evident between the people in the clip ?*

1 2 3 4 5 6 7

None at all

A lot

*Based on the clip you have just watched, please estimate how much the people in the clip* ***enjoy each other’s company***

1 2 3 4 5 6 7

They do not enjoy each other’s company at all

They enjoy each other’s company a lot

*Based on the clip you have just watched, how likely do you think it is that the people in the clip will* ***work together in the future?***

1 2 3 4 5 6 7

Highly Likely

Not at all likely

Study 3

Thank you for agreeing to take part in this research. This study is on social perception. We are interested in how people make social judgments based on limited information.

Usually we make social decisions about other people, but a famous study from the 1950s showed that our social perception skills are very sensitive and can be triggered by simple geometric shapes like triangles and circles.

This might seem strange - how could we make social judgments about triangles? In a moment we will show you the clip used in this famous study. As you watch it, see if you can work out the relationship between the small dot and the large triangle

<heider & simmel clip>

How would you describe small dot?

Would you say it was:

1. Fearful

or

2. Aggressive

<press 1 for Fearful or 2 for Aggressive>

Most people think that the dot in the clip is fearful. Even though they were only simple shapes, most people are able to make a judgment about the social relationship between them. This is what we mean when we say that we are studying how people can make social judgments based on limited information - you probably haven't ever judged the social relationship of two shapes before, but it isn't as difficult as you might think.

We are now going to show you some film-clips like the one you just saw. Each clip shows two circles moving from the left of the screen to the right. As you watch these circles move from one side of the screen to the other, try to imagine what kind of relationship exists between them.

Although this might seem an odd thing to do, try to get a feeling for what their social relationship might be like based on their behaviour in the clip.

We have deliberately made this task difficult because we want to see how people make judgements based on limited information. After each clip, you will be asked some questions about the shapes.

The first of these questions will ask about the PROXIMITY between the two shapes. This means we want you to judge how PHYSICALLY CLOSE to each other the shapes were during the clip. You will be shown a scale that goes from 1 (Low Proximity; the shapes did not stay close to each other) to 7 (High Proximity; the shapes stayed very close to each other)

You will then be asked about how much SYNCHRONY the shapes showed in their behaviour during the clip. This means that we want you to judge how much SIMILARITY there was in the movements of the two shapes during the clip. Some clips will show them behaving in EXACTLY the same way (perfect synchrony) and some clips will show them behaving in completely different ways (no synchrony). Again, you will be shown a scale that goes from 1 (Low Synchrony; the shapes were doing completely different things) to 7 (High Synchrony; the shapes were doing exactly the same things in perfect synchrony).

After this we will then ask you some questions about the nature of the social relationship between the two shapes. Again, your answers will be on a scale between 1 and 7, and the scale will be provided.

There are no right or wrong answers. Just be as honest as you can when reporting your judgments.

Please judge each clip individually and try not to let your judgment be influenced by previous clips

REMEMBER: As you watch the clip, try to imagine the social relationship that might exist between the circles

<clips>

*Based on the clip you have just watched, how much* ***trust*** *do you think exists between the two circles?*

1 2 3 4 5 6 7

They do not trust each other at all

They trust each other completely

*Based on the clip you have just watched, how might you describe the* ***relationship*** *between the two circles?*

1 2 3 4 5 6 7

Like “blood relatives”

Like total strangers

*Based on the clip you have just watched, how would you describe the level of* ***bonding*** *between the two circles?*

1 2 3 4 5 6 7

Not bonded at all

Extremely well bonded

*Based on the clip you have just watched, how much* ***humour*** *do you think exists between the two circles?*

1 2 3 4 5 6 7

None at all

A lot

*Based on the clip you have just watched, how much* ***camaraderie*** *do you think exists between the two circles? (*camaraderie = a spirit of familiarity and trust*)*

1 2 3 4 5 6 7

None at all

A lot

*Based on the clip you have just watched, how much* ***friendship*** *do you think exists between the two circles?*

1 2 3 4 5 6 7

None at all

A lot

*Based on the clip you have just watched, how much* ***rapport*** *do you think exists between the two circles? (*Rapport *=* a close and harmonious relationship in which the people concerned understand each other's feelings or ideas and communicate well)

1 2 3 4 5 6 7

None at all

A lot

*Based on the clip you have just watched, how much* ***co-operation*** *do you think would be evident between the two circles ?*

1 2 3 4 5 6 7

None at all

A lot

*Based on the clip you have just watched, please estimate how much the two circles* ***enjoy each other’s company***

1 2 3 4 5 6 7

They do not enjoy each other’s company at all

They enjoy each other’s company a lot

*Based on the clip you have just watched, how likely do you think it is that the two circles will* ***work together in the future?***

1 2 3 4 5 6 7

Highly Likely

Not at all likely

Study 4

**Instructions**

We are interested in how people think about groups. Specifically, we are studying the perception of social cohesion. Cohesion is how "together" or "bonded" a group is. Groups differ in all sorts of ways, and how cohesive they are is one.

In a moment you will read two short scenarios. Each scenario describes a collection of people. We want you to think carefully about each scenario and answer some questions about how you perceive the social relationships between the members of the group. The scenarios will not provide you with much information. This is deliberate. We want you to use your imagination without going beyond the scenario described.

For example, you might be asked to imagine the following scenario:

**We would like you to imagine a group of 12 people who are a team competing in a sports tournament. The members of the group have known each other for a year.**

When presented with the scenario, we will then ask you to think about it for a couple of moments, before asking you to answer some questions about how you imagined the social dynamics of the group described in the scenario. For example, you might be asked about how friendly the group members are towards each other. You will answer these questions on a 7-point scale (1 = not friendly at all; 7 = Very Friendly).

**It is important that you get a clear sense in your head about what the group described might be like. After reading the scenario, please spend a couple of moments thinking about it and trying to get an idea about the social dynamics of the group.**

One you are ready please continue to the first scenario:

*SCENARIO 1*

We would like you to imagine:

***A group of eight people traveling on an important journey. The journey will take three days. None of the group knew each other before they began the journey. All members of the group volunteered to go on the journey.***

Please take a moment to think about this group. Try to imagine what the group might be like during the three days that they are on the journey.

Take as long as you wish to get a clear idea in your head. Once you have done that, please continue and answer some questions.

**Please do not continue until you have a clear image in your head about what this group might be like.**

Just so we know you’ve been paying attention, please answer the following questions about the scenario that you have just read (it’s fine to scroll back up to look at the description again at any point if you need to)

Based on the information you have been given, which of the following options is the most accurate description of what you have been told the group is going to be doing during its time together?

Traveling on an important journey

Planning an important party

Looking for an important person

Attending an important event

How long will the group be together on their journey?

3 months

3 weeks

3 days

3 hours

Did the members of the group know each other?

Yes

No

How many people were in the group?

4

6

8

10

In the spaces provided, please suggest 2 things that you imagined happening during the time that the group was together.

| 1 |  |
| --- | --- |
|  |  |
| 2 |  |

|  |
| --- |

We will now ask you some questions about how you imagined the social dynamics of the group during the activity described. Please answer these questions by selecting the appropriate number on the scale provided. There are no right or wrong answers so please be as honest and as accurate as you can. Remember to answer based on how you imagined the group during the time they were together. Please do not base answers on anything you might have imagined before or after the scenario described.

Based on the scenario you have just imagined, how much **trust** do you think the members of the group would have for each other during the journey?

1 They would not trust each other at all

2

3

4

5

6

7 They would trust each other entirely

Based on the scenario you have just imagined, how might you describe the **relationship** between group members during the journey?

1 Like total strangers

2

3

4

5

6

7 Like “blood relatives”

Based on the scenario you have just imagined, how would you describe the level of **group-bonding** during the journey?

1 Not bonded at all

2

3

4

5

6

7 Extremely well bonded

How much **humour** do you think would be evident within the group during the journey?

1 None at all

2

3

4

5

6

7 A Lot

How much **friendship** do you think would be evident within the group during the journey?

1 None at all

2

3

4

5

6

7 A lot

To what extent do you think the group find their time together **enjoyable**?

1 Not enjoyable at all

2

3

4

5

6

7 Very enjoyable

Based on how you imagined the scenario, how likely do you think it is that the group would voluntarily spend time together in the **future**?

Not at all likely

2

3

4

5

6

7 Very likely

Based on how you imagined the scenario, to what extent would you say the group members **shared the same goals** while they were traveling?

1 Their goals did not overlap at all

2

3

4

5

6

7 Their goals overlapped entirely

"Common fate” is the extent to which an individual's fate is linked to the rest of the group. Based on how you imagined the scenario, to what extent would you say that the group members **shared a common fate** during the time they spent traveling?

1 The fate of each individual was entirely separate to the rest of the group

2

3

4

5

6

7 The fate of each individual was entirely linked to the rest of the group

You are now about to see the second scenario. As before, please try to get a clear sense in your head of what the group is like, without going beyond the scenario described.

*SCENARIO 2*

We would like you to imagine:

***A group of eight people attending an important event. The event will last for three days. None of the group knew each other before they met at the event. All members of the group volunteered to attend the event.***

Please take a moment to think about this group. Try to imagine what the group might be like during the three days that they are at the event.

Take as long as you wish to get a clear idea in your head. Once you have done that, please continue and answer some questions.

**Please do not continue until you have a clear image in your head about what this group might be like.**

Just so we know you’ve been paying attention, please answer the following questions about the scenario that you have just read (it’s fine to scroll back up to look at the description again at any point if you need to)

Based on the information you have been given, which of the following options is the most accurate description of what you have been told the group is going to be doing during its time together?

Traveling on an important journey

Planning an important party

Looking for an important person

Attending an important event

How long will the group be together at the event?

3 months

3 weeks

3 days

3 hours

Did the members of the group know each other?

Yes

No

How many people were in the group?

4

6

8

10

In the spaces provided, please suggest 2 things that you imagined happening during the time that the group was together.

| 1 |  |
| --- | --- |
|  |  |
| 2 |  |

|  |
| --- |

We will now ask you some questions about how you imagined the social dynamics of the group during the activity described. Please answer these questions by selecting the appropriate number on the scale provided. There are no right or wrong answers so please be as honest and as accurate as you can. Remember to answer based on how you imagined the group during the time they were together. Please do not base answers on anything you might have imagined before or after the scenario described.

Based on the scenario you have just imagined, how much **trust** do you think the members of the group would have for each other during the event?

1 They would not trust each other at all

2

3

4

5

6

7 They would trust each other entirely

Based on the scenario you have just imagined, how might you describe the **relationship** between group members during the event?

1 Like total strangers

2

3

4

5

6

7 Like “blood relatives”

Based on the scenario you have just imagined, how would you describe the level of **group-bonding** during the event?

1 Not bonded at all

2

3

4

5

6

7 Extremely well bonded

How much **humour** do you think would be evident within the group during the event?

1 None at all

2

3

4

5

6

7 A Lot

How much **friendship** do you think would be evident within the group during the event?

1 None at all

2

3

4

5

6

7 A lot

To what extent do you think the group find their time together **enjoyable**?

1 Not enjoyable at all

2

3

4

5

6

7 Very enjoyable

Based on how you imagined the scenario, how likely do you think it is that the group would voluntarily spend time together in the **future**?

Not at all likely

2

3

4

5

6

7 Very likely

Based on how you imagined the scenario, to what extent would you say the group members **shared the same goals** while they were at the event?

1 Their goals did not overlap at all

2

3

4

5

6

7 Their goals overlapped entirely

"Common fate” is the extent to which an individual's fate is linked to the rest of the group. Based on how you imagined the scenario, to what extent would you say that the group members **shared a common fate** during the time they spent at the event?

1 The fate of each individual was entirely separate to the rest of the group

2

3

4

5

6

7 The fate of each individual was entirely linked to the rest of the group

We would now like to ask you some questions about your experience of taking this survey. Please respond truthfully – your accurate response here helps us make sure that our research is reliable! Your answers will NOT affect whether or not you receive your credit, so please be honest.

*How distracted did you feel as you completed this survey?*

1 Not at all

2

3

4

5

6

7 Very Distracted

*Did you read both of the scenarios properly and think about them as instructed?*

Yes

No

*To what extent did you hurry through this survey?*

1 Not at all

2

3

4

5

6

7 Very Much

Is there anything else that you think we should know?
