# Supplementary material for: Collective directional movement and the perception of social cohesion
Source: Br J Soc Psychol. 2020 Jan 3;59(4):819–38. doi: 10.1111/bjso.12361 (PMC7586976; doi:10.1111/bjso.12361)
Supplement: Supplementary file 2 — Code S1. Materials. [file BJSO-59-819-s002.docx]

CODE

**Study 1 Code:**

InterceptOnly Model: = glm(formula = cohesion ~ 1, data = myData)

Model 1 = lmer(cohesion ~ 1 + (1|id), data = myData, REML=FALSE)

anova(Model 1,InterceptOnly Model)

Model 2 = lmer(cohesion ~ movement + (1|id), data = myData, REML=FALSE)

anova(Model 2, Model 1)

Model 3 = lmer(cohesion ~ activity + (1|id), data = myData, REML=FALSE)

anova(Model 3, Model1)

Model 4 = lmer(cohesion ~ activity + movement + activity*movement + (1|id) , data = myData, REML=FALSE)

anova(Model 4, Model2)

**Study 2 Code:**

InterceptOnly Model: glm(formula = cohesion ~ 1, data = myData)

Model 1 = lmer(cohesion ~ (1|stimulus) + (1|id), data = myData, REML = FALSE)

anova(InterceptOnly, Model 1)

Model 2 = lmer(cohesion ~ condition + (1|stimulus) + (1|id), data = myData, REML = FALSE)

anova(Model 1, Model 2)

**Study 3 Code:**

InterceptOnly Model: glm(formula = cohesion ~ 1, data = myData)

Model 1 = lmer(cohesion ~ (1|stimulus) + (1|id), data = myData, REML = FALSE)

anova(InterceptOnly,Modle1)

Model 2 = lmer(cohesion ~ synchrony + (1|stimulus) + (1|id), data = myData, REML = FALSE)

anova(Model1,Model2)

Model 3 = lmer(cohesion ~ proximity + (1|stimulus) + (1|id), data = myData, REML = FALSE)

anova(Model1,Model3)

Model 4 = lmer(cohesion ~ proximity + synchrony + (1|stimulus) + (1|id), data = myData, REML = FALSE)

anova(Model 2, Model 4)

anova(Model 3, Model 4)

Model 5 = lmer(cohesion ~ proximity + synchrony + proximity*synchrony + (1|stimulus) + (1|id), data = myData, REML = FALSE)

**Study 4 Code:**

Intercept Only Model = glm(Cohesion ~ 1, data = data)

Model 1 = lmer(Cohesion ~ 1 + (1|ID), data = data, REML=FALSE)

anova(Model 1, Intercept Only Model)

Model 2 = lmer(Cohesion ~ Condition + (1|ID), data = data, REML = FALSE)

anova(Model 1, Model 2)

Model 3 = lmer(Cohesion ~ Condition + Fate + (1|ID), data = data, REML = FALSE)

anova(Model 2, Model 3)

Model 4 = lmer(Cohesion ~ Condition + Fate + Condition*Fate + (1|ID), data = data, REML = FALSE)

anova(Model 3, Model 4)

Model 5 = lmer(Cohesion ~ Condition + Goals + (1|ID), data = data, REML = FALSE)

anova(Model2,Model5)

Model 6 = lmer(Cohesion ~ Condition + Goals + Goals*Condition + (1|ID), data = data, REML = FALSE)

anova(Model 5,Model 6)

Model 7 = lmer(Cohesion ~ Condition + Goals + Fate + (1|ID), data = data, REML = FALSE)

anova(Model 5,Model 7)

anova(Model 3,Model 7)

Model 8 = lmer(Cohesion ~ Condition + Goals + Fate + Goals*Fate + (1|ID), data = data, REML = FALSE)

anova(Model 8,Model 7)

Model9 = lmer(Cohesion ~ Goals + Fate + (1|ID), data = data, REML = FALSE)

anova(Model 7,Model 9)

Model 10 = lmer(Fate ~ 1+ (1|ID), data = data, REML = FALSE)

Model 11= lmer(Fate ~ Condition + (1|ID), data = data, REML = FALSE)

anova(Model 10,Model 11)

Model 12 = lmer(Goals ~ 1+ (1|ID), data = data, REML = FALSE)

Model 13= lmer(Goals ~ Condition + (1|ID), data = data, REML = FALSE)

anova(Model 13,Model14)
